# Optimising Repeated Exposure: Determining Optimal Stimulus Shape for Introducing a Novel Vegetable among Children

**DOI:** 10.3390/foods10050909

**Published:** 2021-04-21

**Authors:** Klelia Karagiannaki, Christian Ritz, Ditte Søbye Andreasen, Raphaela Achtelik, Per Møller, Helene Hausner, Annemarie Olsen

**Affiliations:** 1 Section for Food Design and Consumer Behaviour, Department of Food Science, Faculty of Science, University of Copenhagen, Rolighedsvej 26, 1958 Frederiksberg C, Denmark; klelia.karagiannaki@outlook.com (K.K.); dittesa@hotmail.com (D.S.A.); kiwiela@googlemail.com (R.A.); p2moller@gmail.com (P.M.); helene.hausner@gmail.com (H.H.); 2 Department of Nutrition and Exercise Science, Faculty of Science, University of Copenhagen, Rolighedsvej 25, 1958 Frederiksberg C, Denmark; ritz@nexs.ku.dk

**Keywords:** repeated exposure, children, vegetables, taste, preferences, shape

## Abstract

Although it is well evident that a healthy diet rich in fruit and vegetables could prevent a number of major chronic diseases, national and international guidelines concerning their intake are not being reached by a large percentage of the population, including children. Thus, it is of interest to investigate how the consumption of this food group by children could be increased. The aim of this study was to examine the impact of serving style on the consumption of a raw snack vegetable (daikon) and the influence of its exposure on liking and intake of the vegetable. A group of 185 children 3–5 years old participated in the study. Two kindergartens served as intervention groups, while the third was assigned to be the control group of the study (*n* = 50). The intervention groups were repeatedly exposed to one of three different serving styles of daikon: sticks (*n* = 42), triangles (*n* = 46) or grated (*n* = 47), and they were all visited 7 times during the exposure period, on the same frequency (twice per week). Familiarity and liking of the target vegetable, daikon, and six other vegetables (cucumber, celery, celeriac, broccoli, cauliflower and beetroot) were measured at baseline, post-intervention and two follow up sessions (3- and 6-month) to investigate the likelihood of generalisation effects. Intake of daikon was measured at all control sessions and exposures. Moreover, children were asked to rank their favourite serving style of daikon and beetroot, among triangle, stick and grated, towards understanding the influence of shape on the efficacy of the exposure. The results revealed significant changes between liking and intake of daikon for the groups of triangles and sticks and the control group (*p* < 0.05). The group that received grated daikon did not show significant differences in liking and at intake levels during the exposures but performed well in the long-term. Throughout the exposure period, intake levels followed an overall increasing pattern, with all the groups to demonstrate a decrease of their intake at the last session, which was not found significant for the triangle group. Mere exposure was efficient towards increasing liking and intake of the novel vegetable with all the shapes to deliver positive results, but based on this study no particular serving style can be recommended.

## 1. Introduction

It is evident that high intake of fruit and vegetables is correlated with a lower possibility of the development of a non-communicable disease [1,2,3,4]. Fruits and vegetables carry a combination of different nutrients, such as vitamin C and other antioxidants, dietary fibre and phytochemicals, which seemingly particularly promote health, and their systematic consumption is negatively correlated with risk factors of cardiovascular disease and some types of cancer [5]. Nordic Nutrition Recommendations 2012 [6] suggest that dietary patterns high in vegetables, fruits, nuts, legumes, fish, vegetable oil and low-fat dairy products (such as Mediterranean-like diets) are associated with a decreased risk of chronic diseases and the prevention of weight gain. The proposed amount for children 4–10 years old is 300–500 g of fruit and vegetables every day, with half of this quantity to be covered from vegetables [6,7]. In Denmark, the consumption of this food group by children is not meeting the recommendations; a daily average of only 84 g of vegetables and 157 of fruits are consumed by 11-year-olds [8].

It has been pointed out that adults preserve eating habits and food preferences acquainted in childhood [9,10,11,12,13] and thus it is important to make changes in young children’s eating habits in order to obtain and maintain a healthy lifestyle throughout the stages of life. Taking the above into account, there is still a need for techniques able to enhance fruit and especially vegetable intake by children.

Children’s acceptance and intake of vegetables is strongly affected by their preference [14,15,16,17,18,19,20], which is defined as choice of one food item over another [21]. Other determinants include liking, accessibility, availability, convenience, appearance and texture of the food stimuli [19,22], as well as peer influence and modelling [23,24].

Vegetables are not much preferred by the majority of children [25] as most of them are bitter and have low energy content. This can be explained as human’s innate food preferences are based on sweet and salty flavours and are affected by neophobia and by an association with the whole meal experience [21]. It is a well-known fact that most food preferences are learned, thus it is obvious that they can be changed [21,26]. The contact of the foetus with the amniotic fluid, as well as early eating experiences after birth, seem to affect the formation of eating habits in childhood [27,28,29]. According to Soldateli et al., preschool children who were breastfed for 12 months or longer showed significantly higher vegetable consumption [30], in line with the findings of Kheir et al. [31]. Research findings show that young children are an easier target for such a procedure in order to achieve healthier food choices [32,33]. According to Harris and Mason [34], the acceptance of new tastes is possible to be learned also later in life, as long as there is motivation.

There are several theories explaining the way we obtain preference to foods and the possibility of changing them. Food preferences are able to form and changed through experience, which involves several procedures. Experience is the main quality through which we form food preferences, while conditioning is a process that contributes to changing them. The latter is about the acquisition of relations between a food’s sensory characteristics and the physiological or hedonic outcome of consuming it. Conditioning is branched in three categories: flavour-nutrient learning, flavour-flavour learning and exposure [21,35,36]. Exposure is a condition getting the stimulus approachable to an individual’s perception [35]. Change in food preference by this mechanism is possible; via exposure or repeated tasting of an initially disliked food, this can be eventually liked or even preferred among others [21,37,38,39,40,41] with the optimum number of exposures to differ between children’s age groups [38]. Repeated exposure to a food may improve familiarity, another important factor which seems to contribute to the forming of food preferences [38] and suppress food neophobia [42,43,44].

Availability is an important aspect shaping the consumption of fruit and vegetables [14,17], while it can also be a driver for the acceptance of a novel food [45]. Krølner et al. [46] showed that fruit and vegetables are not adequately available outside the home in many countries. The term of *accessibility* defines if foods are available in an arrangement that make their consumption easy [17]. Several studies have showed that increased accessibility can improve intake of fruit and vegetables by children [14,16,17,46,47].

An additional factor affecting children’s preferences is the comfort of obtaining, preparing, transporting and/or eating of food, which can be described as convenience. It is related with less healthy products, as it was found that fruit and vegetables are considered by children as inconvenient even if they are liked, because of the time necessary for their preparation [46].

Texture seems to be also important [48] as young children often exclude foods due to their texture if it is difficult to handle in the mouth [22,49]. It has been shown that children prefer fresh, raw vegetables over cooked as they are more crispy, crunchy and juicy [46,49], while this seems to depend also on cultural parameters [50].

*Food neophobia* has been described as the rejection of food on sight [51], a fact that indicates that appearance plays an important role in food acceptance. According to Brown and Harris [52], this can be explained because of the innately forming of conclusions about a food’s safety according to its looks. Food in general triggers humans visually first, stimulating the cephalic phase responses [53], with the visual appearance—including size, colour and shape—to potentially urge expectations concerning the other sensory characteristics of the stimuli [54,55]. Therefore, altering this attribute may boost acceptance and consumption of unfamiliar foods, which is of high importance as food neophobia is connected with poor quality of diet and adverse changes of health-related biomarkers and risk factors that are linked to non-communicable diseases [56]. It has been shown that children give more attention to visual appearance in foods than adults [57] and it is quite interesting that Hill et al. [58], as well as Zeinstra et al. [19], showed in their studies that appearance is more important for disliking vegetables particularly for younger children than for older ones. This is completed by the fact that the stage of neophobia peaks between the 2nd and the 6th year of the child [51,59,60,61,62]. Colour, size and shape are the major appearance attributes. Harris highlights that “small and seemingly irrelevant” details may be definitive for the acceptance or rejection of the food [48]. Maratos and Staples [63] found that the visual aspect of food plays a crucial role for children in trying novel foods, especially when they are neophobic, as vision seems to be neophobia’s first principal domain. For Dovey et al. [64], appearance of food was perceived as the most important predictor for 5–10-year-olds’ consumption of a novel fruit. This is in line with Dazeley et al. [65] and Coulthard et al. [66], who suggest that improving visual appearance has the potential to increase liking and acceptance.

Although it might be apparent that the visual cues of vegetables can be easily modified by cutting them in different shapes, there is a need for more literature on this matter. Bönnhoff et al. [67], proposed that cutting and serving fruit and vegetables ready-to-eat is able to increase the intake in 7–10-year-old children by enhancing accessibility. Another study which examined several factors of visual appearance and serving style of raw snack vegetables in correlation with their liking, found out that children 9–12 years old were affected by serving style, as they preferred more cut over uncut vegetables, especially those cut in special shapes [68]. Kranz et al. [69] indicated that children-friendly shaped fruit and vegetable snacks were perceived as being more “fun” than the regular-shaped ones and were more likely to be selected. These results are in agreement with the pilot study of Chung and Fong [70], who discovered that a combined approach of repeated exposure along with alterations in the appearance of initially disliked vegetables can improve 7–10 year-old children’s inclination to try.

It had been also discussed in research the fact that people are affected by their eating companions on the quality and the quantity of the food consumed [21,71], as well as by the eating attitudes of the familial environment, something that is especially true for children [28,72,73,74].

It is important to highlight that culture and tradition play an important role towards familiarisation and acceptance of foods, as they affect the attributes that determine rejection. In the cross-cultural study of Sandvik et al., it was demonstrated that drivers of liking of a high-fibre biscuit were more or less the same in terms of taste, but quite variable concerning texture, and especially crunchiness, which was found as a “must-have” for Swedish children [50]. Similarly, the study of Estay et al. found significant differences in the hedonic score of 6 common vegetables (broccoli, corn, cucumber, mushrooms, potatoes and sweet peas) between children from China, Chile and the US, with Chinese children to show overall higher acceptance [75].

### Aim of the Study

This study aimed to examine the impact of serving style on consumption of a novel snack vegetable among 3–5-year-old children by use of repeated exposures. The study was part of a larger study [76], which moreover investigated the effect of exposure frequency to the consumption of a novel vegetable, sharing some experimental parts and groups.

## 2. Materials and Methods

### 2.1. Study Design

A total of three kindergartens with eight groups and 212 children participated. Two kindergartens served as intervention kindergartens and the groups were randomly assigned to the various shapes. All experimental groups conducted a baseline and a postintervention test, which measured familiarity, liking and preferences of the target vegetable and six other vegetable stimuli (cucumber, celery, celeriac, cauliflower, broccoli and beetroot) as well as the intake of the target vegetable stimulus. The post-test was completed approximately one week after the last exposure in the intervention kindergartens and six weeks after the pre-test in the control kindergarten. Three- and six-month follow-ups, similar to the post-test, also took place. The details of the different visits to the kindergartens are given in Table 1.

The three groups from each of the two intervention kindergartens were randomly assigned to a shape type: daikon cut in sticks, triangles or grated, and during the exposure sessions, children were invited to eat as much or as little as they wanted. All groups received a vegetable serving of 100 g. This procedure was repeated for seven exposure visits, scheduled twice per week, only for the three intervention groups. This exposure frequency to the vegetable was the same for all serving styles. The outcomes measured before and after the intervention were for liking and intake of the target stimulus (daikon) by recording the vegetable intake in g.

The control kindergarten was also divided in two groups, which did not undergo any exposures but were just visited at baseline, post intervention, with a 3- and 6-month follow up.

### 2.2. Recruitment and Participants

Local kindergartens in Copenhagen were invited to participate. The parents provided written, informed consent, and it was voluntary for the children to participate. After a review of the study protocol by the Danish National Committee on Biomedical Research Ethics, it was concluded that a formal ethical approval was not required (ref. H-2-2011-FSP9). A gift certificate worth DKK 50 (EUR ~7) to a popular Danish toy store was used to incentivise parents to return the questionnaire.

### 2.3. Stimuli

Seven different vegetables were used for the measurement of familiarity and liking at baseline, post intervention and the follow-ups: daikon, cucumber, celery, broccoli, cauliflower, celeriac and beetroot. Only daikon and beetroot were examined as far as different shapes and texture were concerned. Daikon was chosen to be the target stimuli, cut in three different shapes: sticks, triangles and grated. This vegetable was selected as it was considered a non-familiar and less-liked vegetable [77], but also because it can be easily cut into different shapes. Moreover, it was rated “medium-liked” in the small pilot study that took place, thus it would be easy to measure the potential increase in liking of this vegetable after the intervention. The target vegetable was served as an afternoon snack in the day-care institution of the children, where the study took place.

Four other vegetables (broccoli, cauliflower, celeriac and beetroot) were chosen as reference vegetables, because they share at least one stimulus component with daikon. Daikon (also termed Chinese radish) is a member of Brassicaceae family, the cabbage family [78]. It has in common several aroma compounds with broccoli and cauliflower, which are in the same family [79]. Cauliflower, celeriac and daikon have comparable white colour, while beetroot and daikon have mutual shapes that they can take (sticks, triangle and grated). Beetroot and celeriac are members of two different families that are not related to the cabbage family [80]. The above shared characteristics were used for generalisation effects testing; to investigate if an increase in the liking of daikon could affect liking of the others, similar to it, vegetables.

Cucumber and celery were chosen as “dummy” vegetables to help children get used to the testing procedure and the 3-point smiley scale. This selection was made considering cucumber as a liked and familiar vegetable and celery as an unfamiliar and disliked one [68]. Both vegetables were served as pieces of 5 cm length, in a style supposed to be familiar with children.

All the vegetables in this study were served raw, as it is proposed in previous research that Danish children are familiar with this style of serving vegetables at breakfast, lunch and in between meals [81,82]. The daikon used for the pre- and post-test to measure intake was served in round slices of a diameter approximately 4.5 cm, while the same vegetable used for the exposure days was cut into sticks (7 cm length), triangles (4.5–5 cm length) or grated depending on the intervention group. These stimuli are categorised as fresh-cut produce; a term to describe the vegetables or fruit that have been altered by trimming, peeling or cutting, remaining in a fresh state and offering a high nutrition. Changes in quality, colour, texture, flavour and nutritional value are a natural consequence when cutting the fresh vegetable in different shapes, with grated daikon being the most sensitive form to those changes as well as the most probable to undergo a faster microbial spoilage and contamination, leading to a decreased shelf life, due to the higher surface area [83].

### 2.4. Measurement of Outcomes

To measure liking and preference, a 3-point facial hedonic scale labelled with the descriptors “like”, “ok” and “dislike” was used for this study, as recommended for use with 3-year-old children [84] and used in previous research [85,86,87,88,89]. Familiarity was measured by asking each child on whether or not they had tasted the specific vegetable before.

### 2.5. Pilot Study

A small pilot study was conducted approximately one month before the kindergarten intervention in order to test the suitability of the selected stimuli as well as the duration of the testing procedure. A group of 16 children participated at the age of 3–5 years. The pilot study verified the usage of daikon as a target vegetable, as it was rated with a mean liking of 2.1 (medium-liked), and the chosen shapes were found to be appropriate for use in the intervention groups.

### 2.6. Intervention Study

All vegetables were delivered by K.C. Frugt and stored at 5 °C. The different shapes were already processed (sliced, as sticks, as triangles and grated) but they were additionally slightly modified in order to achieve a perfectly round shape, while they were checked for brown colouring. The pre- and the post intervention test were operated in the morning—between 9.00 and 11.00—thus some samples were prepared the previous day, while the exposure visits took place in the afternoon and the samples were prepared the same morning. A quiet area of the kindergarten was chosen in order to provide a familiar and comfortable environment for the children during the tasting sessions.

#### 2.6.1. Test Sessions

Test sessions were separated into two parts: individual testing and assessment of intake. The individual testing took place on a one-to-one basis with one child per assistant. Children were instructed on how to use the scale as well as to just taste the vegetable and not eat it all during the tasting procedure. Familiarity was tested by asking the children if they had tasted the vegetable in question before. When liking was measured, the assistants encouraged the children to taste a piece of the vegetable and to indicate their liking by pointing to a smiley face on the scale. The same procedure was repeated for all seven vegetables, with the use of water as a palate cleanser in between of each sample. The dummy vegetables (cucumber and celery) were presented first, while beetroot and daikon were always served last. The order of the other reference vegetables was randomised by the assistants and noted down on the questionnaire.

The children were served a slice of each of the seven vegetables towards evaluating liking, and then their preference was measured for the three different shapes of beetroot and daikon. The three different shapes (triangle, grated or sticks) of beetroot or daikon, according to the random order of serving, were presented to the child on a plate, requesting to taste all three styles and select the one he/she liked the best. After the selected one was removed by the assistant from the plate, the child was asked to rate the remaining two styles without allowing any ties. This procedure was completed for the remaining vegetable (daikon or beetroot). Water was used as a palate cleanser in between the different stimuli. In case of refusal of a child to taste a vegetable, there was tried to rank the three styles only by appearance, without forcing the child to try.

Intake was measured in the control sessions by weighting the individual amount of vegetable left for every child using a precision scale (Sartorius Gram TE15025, Sartorius AG, Göttingen, Germany). Children were invited to eat as much, or as little, as they wanted, and they could also be served with another 100 g of the vegetable if they wanted.

#### 2.6.2. Exposure Sessions

The six intervention kindergarten teams were visited seven times during the intervention. Children received a small plastic beaker with daikon in sticks, triangles or grated, according to each intervention group. The grated daikon was served along with a child-sized fork, and headspace in serving beakers were refreshed to minimize smell. During the exposure days, children received 100 g of the vegetable but not the possibility of a second serving, unlike the control sessions where the maximum intake would be 200 g. Those visits were completed in the afternoon and children were served the vegetable as their afternoon snack at around 14.30.

#### 2.6.3. Control Group

Children in the control group completed the four tests and were thus visited eight times in total. Measurement of liking, familiarity and preference was achieved with the same process described earlier and measurement of the intake of daikon was completed some days after the pre-test. Daikon was served in slices. Intake of the target vegetable was measured again six weeks later, and the post-test was conducted after a few days.

### 2.7. Questionnaire

A questionnaire was given to all parents of the children in the three kindergartens with questions related to the child participating in the study, parent’s socioeconomic status, height, weight and fruit and vegetable intake. Besides demographics, the questionnaire included questions on the child’s fruit and vegetable intake, the familiarity and liking of the stimuli used in the study and the general availability of this food group at home. Some questions by the Comprehensive Feeding Practices Questionnaire (CFPQ) were included, concerning dietary rules and feeding practices [90], as well as the Children’s Eating Behaviour Questionnaire (CEBQ) to cover child’s eating behaviour [91]. In addition to those, six questions assessing food neophobia were included (adapted version of Pliner [92]), as well as some questions about accessibility and serving style and one question on child’s textural preferences. The questionnaire is available from the authors upon request.

### 2.8. Sensory Profiling Method

The vegetables used in the study were sensory described with the application of a Quantitative Descriptive Analysis method (QDA) modified from the ordinary by giving the panel a predetermined set of attributes. The aim of this method was to obtain an accurate sensory description of the vegetables and clearly distinguish how those vegetables differ.

Two preliminary tastings of the vegetables took place, and it was decided to apply sensory descriptive analysis on 9 of 13 in total samples that were served in the kindergarten, excluding cucumber, celeriac, beetroot stick and triangle. The samples were prepared the same way with the ones for the kindergarten intervention. An amount of 100 g of each vegetable was served, same with the kindergarten intervention. The samples were stored at 4 °C and one hour prior to the serving they were taken out of the refrigerator. According to Lawless and Heymann [93], consensus within the panel and reproducibility for each panellist are better controlled when the samples are in triplicates, and thus it was decided to do the same for this study. The samples were coded with random three-digit numbers [94] and each replicate had a different code [95]. A 15 cm line scale with indented anchors was used for rating the samples. This procedure was completed for all the samples on three consecutive days in order to minimise sensory fatigue. Unsalted crackers and tempered water (40 °C) followed by cold water were chosen for the palate cleansing, as daikon’s pungent taste can be difficult to eject.

The panel underwent a training of two 30-min sessions each on two consecutive days. The first training session included the presentation of all nine samples and 21 attributes to describe them, the meaning of each was explained to the panel. Next, the panel had to get familiar with the samples by looking at them, smelling and tasting them. The third part of this session concerned the description of grated and triangles of daikon, served in 100 g, using all attributes. During the second training session, the members of the panel received a modified score sheet with seven attributes that, according to the first session, found out to be the most difficult ones to score. The panel was asked to score grated daikon, broccoli and daikon sticks concerning texture. In the final evaluation, all nine samples were served together with an instruction sheet, score sheets, crackers, 40 °C tempered water, cold water, pen, napkin, fork and spittoon.

### 2.9. Data Analysis

The data were analysed using the open-source software RStudio (Version 1.2.5019 © 2009–2019 RStudio, Inc, Boston, MA, USA), while Microsoft Excel (Version 16.26 (20091400) © 2020 Microsoft Corporation, Redmond, WA, USA) was used to structure the data, calculate the Standard Deviation (SD) and Standard Error of the Mean (SEM) and prepare the plots. For the children of the exposure groups, an exclusion criterion was implemented; a child was excluded from the data analysis if he/she has participated in less than four of the seven exposure sessions.

The correlation between liking of daikon and other vegetables with the different serving styles was analysed using the R packages *nlme* [96] and *multcomp* [97]. *nlme* was used to run the linear mixed model (*lme function*) and *multcomp* to calculate the difference of the differences between and within the intervention and control groups for each session (*glht function*). The model used included fixed and random factors and an interaction term for serving style and timing. The fixed factors were baseline daikon liking, baseline familiarity to daikon, age and gender, and the random factors were the participants’ ID number, group and institution. The model was used without any reductions.

The intake of daikon during the control sessions (pre-test, post-test, 3-month follow up and 6-month follow up) was associated to the various serving shapes using the same R packages mentioned above, *nlme* and *multcomp*. The linear mixed model was run using the *function lmer*, while *glht function* was used to calculate the difference of the differences and extract the *p*-values. This model was used without reduction in the analysis, and it contained both fixed and random factors, as well as an interaction term for serving style and timing. The fixed factors were baseline daikon liking, baseline familiarity to daikon, age and gender, and the random factors were participants’ ID number, group and institution. The average intake of daikon during the seven exposure sessions was analysed similarly.

The ranking data were analysed using the same R packages and functions, following the assumption that the data is continuous in order to perform the parametric tests. This was according to the central limit theorem (CLT), which suggests that when we have a population with a mean μ and standard deviation σ, and the samples taken are sufficiently large, the distribution of the sample means will have a more or less normal distribution [98]. The initial model included as fixed factors age and gender, and it was reduced as these factors were not shown to be significant.

*p*-values were taken from RStudio from the outputs of *glht function*.

#### Sensory Profiling Data

The sensory profiling data were obtained from the scales using a ruler by hand and were inputted in Excel for the structure and the calculation of means and the Standard Error of the Mean (SEM). The open-source PanelCheck (version 1.4.0) software was used to analyse the performance of the panel, while the sensory data was analysed by Principal Component Analysis (PCA) using The Unscrambler X 10.2 (Camo Analytics, Oslo, Norway). Two- and three-way ANOVAS were used for the panel performance, to cover the replicate effect. All data was full cross-validated, standardised and mean-centred.

## 3. Results

### 3.1. Participant Characteristics

A total of 27 of the 212 children who took part in the study were excluded from the analysis as they were present in less than four exposures, yielding 185 children for the data analysis. Age and gender distribution were different in the four groups, as demonstrated in Table 2, but in the analyses no significant age or gender effect were found.

The response rate for the questionnaires given to the parents was very low (51.2%), so the data obtained were not included in the analyses.

### 3.2. Liking

**Figure 1:**
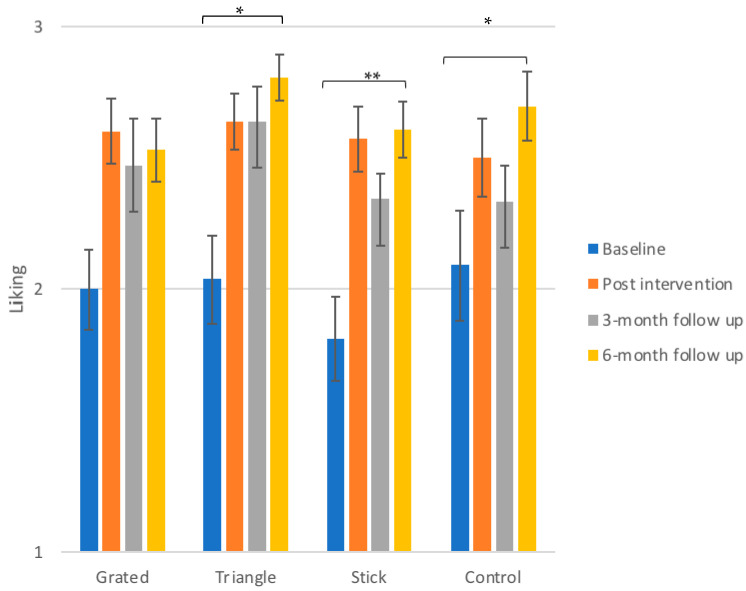
Mean liking scores ± SEM of daikon for the three different exposure groups in the control sessions. The significance of the overall differences only (baseline to 6-month follow up) for the groups of triangles, sticks and the control group are depicted. The overall increase in liking of daikon was not found significant for the group that received grated daikon. The detailed differences and their statistical significance are given in text. Significance levels: ** *p* ≤ 0.01, * *p* ≤ 0.05.

Figure 1 illustrates mean liking scores between and within groups during the four test sessions (baseline, post intervention, 3-month follow up and 6-month follow up). Overall, liking is increasing throughout the study for all groups, with the stick, triangle and control group showing significant changes.

#### 3.2.1. Control Group

Liking for daikon increased from baseline to post intervention from 2.0 ± 0.2 to 2.5 ± 0.2, although this increase was not statistically significant. A drop was observed at the 3-month follow up (2.3 ± 0.2), but the liking rose at 6-months reaching its highest for the control group (2.7 ± 0.1), a value that is significantly elevated compared to baseline (*p* ≤ 0.05).

#### 3.2.2. Intervention Groups

For the group that received grated daikon, no statistically significant changes were found, although liking increased from 2.0 ± 0.2 of baseline to 2.5 ± 0.1 at 6 months. The children that were served with daikon cut in triangles improved their liking significantly from baseline to the 6-month follow up (from 2.0 ± 0.2 to 2.8 ± 0.1, *p* ≤ 0.05). The liking score of this group at 6 months was the highest liking value recorded in this study. Sticks were shown to be a shape that enhanced the liking of daikon from earlier on, expressing significant changes between baseline and all the following control sessions. It should be noted here that the initial liking of daikon for this group was the lowest among the children, equal to 1.8 ± 0.2. This increased to 2.6 ± 0.1 post intervention (*p* = 0.004), dropped slightly to 2.3 ± 0.1 at 3 months (*p* = 0.02) and elevated again to reach 2.6 ± 0.1 at 6 months (*p* ≤ 0.01).

In all groups, including control, the liking score was ameliorated at 6-month follow up, with the group that received daikon cut in triangles had the highest liking value while the children that received daikon cut in sticks followed a more systematic pattern of liking increase. It is interesting to observe that while liking was increasing for all groups from baseline to the post-test, for the majority of the groups (control, grated and stick) the liking at the 3-month follow up dropped before elevating again at 6 months, although these drops were not found statistically significant. For the triangle group we observed that the liking at the 6-month point remained stable, indicating that this shape might be promising for a more concrete and steady improvement of liking of the vegetable.

### 3.3. Intake

**Figure 2:**
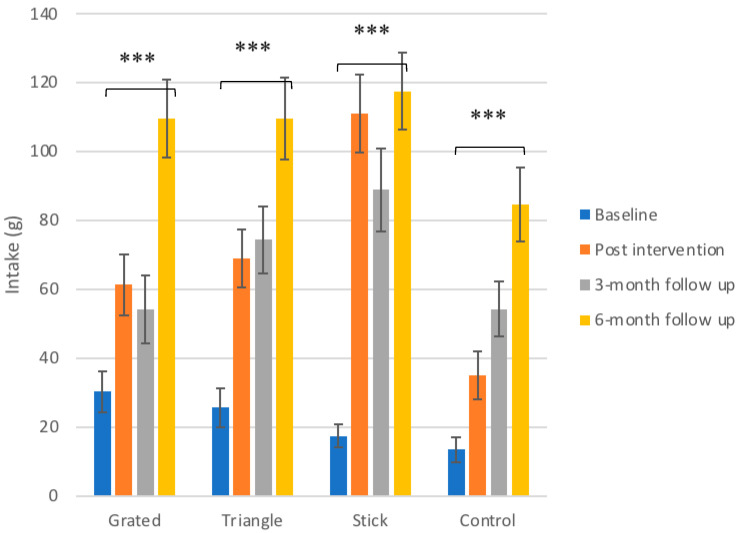
Mean intake scores of daikon ± SEM in g for the three different exposure groups in the control sessions. The significance of the overall differences only (baseline to 6-month follow up) for all groups are depicted. The detailed differences and their statistical significance are given in text. Significance levels: *** *p* ≤ 0.001.

Intake levels at the control sessions are illustrated in Figure 2. The results from the statistical model investigating the impact of the exposure on different serving styles to intake of daikon showed that the latter increased overall from baseline to 6-month follow up significantly for all the groups. The intake rise fluctuated from 70–100 g with the lowest value to represent the control group and the highest the group of sticks.

#### 3.3.1. Control Group

The children in the control group had the lowest initial intake of daikon, equal to 13 ± 3 g, which elevated to 35 ± 7 g at post-test, a change that was found significant (*p* ≤ 0.05). The intake of daikon continued to rise for the control group to 54 ± 8 at 3 months (*p* ≤ 0.001) and 85 ± 11 at 6 months (*p* ≤ 0.001). The control group followed a steady, increasing trend even though it received infrequently small quantities of daikon.

#### 3.3.2. Exposure Groups

For the group that received grated daikon, intake increased from 30 ± 3 g at baseline to 61 ± 5 g at post intervention. Intake decreased at 3-month follow up (54 ± 5 g) but reached its highest level at 6-month follow up (110 ± 6 g). The differences between baseline intake and post intervention (*p* ≤ 0.01), 3-month follow up (*p* ≤ 0.05) and 6-month follow up (*p* ≤ 0.001), as well as the difference between post intervention and 6-month follow up (*p* ≤ 0.001) were found statistically significant for this group.

The children that received daikon cut in triangles elevated the baseline intake from 26 ± 6 g to 69 ± 8 g post intervention and then 73 ± 10 g at 3 months and finally 110 ± 12 g at 6 months, with the change between post-test and 6-month follow up found to be statistically significant (*p* ≤ 0.001). This group also followed a continuously rising trend in intake.

The children who were served daikon cut in sticks demonstrated a rapid and large increase in intake, from 17 ± 3 g of baseline to 111 ± 11 g post intervention (*p* ≤ 0.001). At 3-months the intake slightly decreased to 89 ± 12 g but reached a peak at 6 months (118 ± 11 g), which was the highest intake among all groups. All the differences between baseline intake and the following sessions were found significant (*p* ≤ 0.001 for the difference between baseline and 6-month follow up).

For the groups that were exposed to grated and cut in sticks daikon, it was observed that similarly to the liking scores, there was a slight drop of intake at 3-month follow up before the value elevated again at 6-months. This decline was significant only for the group that received daikon cut in sticks, with the intake at 3-month follow up to be significantly lower than the one post intervention (*p* ≤ 0.05) and the intake at 6-month follow up to be significantly higher than the one at 3 months (*p* ≤ 0.05). Besides this drop, this group reached the highest intake at 6 months among all groups. In comparison, the triangle group demonstrated again a steady increase pattern, similarly to the liking scores.

### 3.4. Exposure Visits

**Figure 3:**
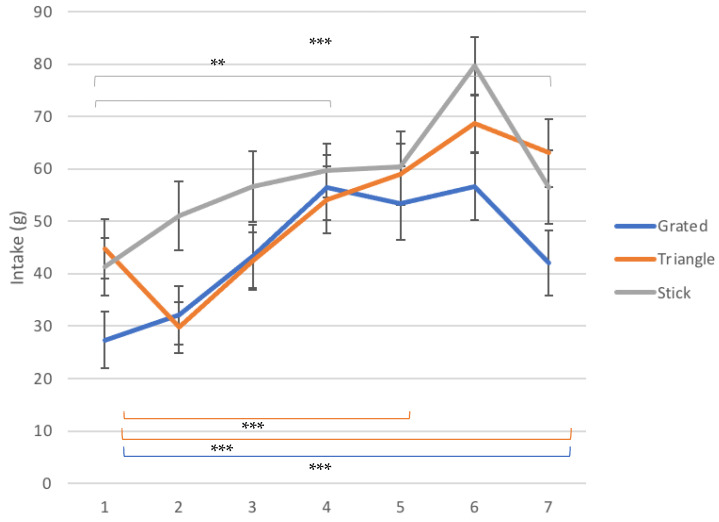
Mean intake scores ± SEM in g of daikon at the different exposure sessions for the three exposure groups. The significance of the differences between selected sessions are depicted for all exposure groups. The group that received daikon cut in triangles and sticks showed a significantly higher intake at the 7th exposure compared to the 1st one, while for the group that received grated daikon the intake at the final exposure was not significantly higher than the initial one. The detailed differences and their statistical significance are given in text. Significance levels: *** *p* ≤ 0.001, ** *p* ≤ 0.01.

Figure 3 illustrates the intake levels of daikon as recorded throughout the exposure period, for the different serving styles. Overall, one can observe that all three groups show an increasing trend in intake, while they all show two timepoints that intake drops, in different exposure session for each group. The highest intake was obtained by the group that was served daikon in sticks and it is equal to 80 ± 6 g. This intake was recorded at the 6th exposure session, right before it dropped to 57 ± 7 g at the 7th exposure.

The triangle group demonstrated the peak of intake at the 7th exposure among the three groups (63 ± 6 g), which was found to be significantly higher than the intake on behalf of the group that was exposed to grated daikon at the same session (*p* ≤ 0.001). The difference between the 7th exposure intake of the stick and triangle group was not significant. Exposure to grated daikon seemed to obtain the lowest intake scores overall throughout the intervention period, which seemed to change in the 6-month follow up as intake then reached levels comparable to the triangle group (110 ± 6 g). Looking at Figure 3, we can observe the affinity of the intake progress that the groups of grated and triangle daikon are demonstrating, remaining for most of the intervention period in similar intake levels, although the grated group follows a declining trend after the 4th exposure session, while the triangle continues to increase. The group that received daikon in sticks achieved overall higher levels of daikon intake throughout the whole intervention, except for the last exposure.

For the group with grated daikon, the intake on the 1st exposure session was found significantly different with the ones at the 3rd (*p* ≤ 0.01), the 4th (*p* ≤ 0.001), the 5th (*p* ≤ 0.01) and the 6th exposure session (*p* ≤ 0.001). Similarly, significantly different were found the changes between the intake at the 2nd exposure and the ones at the 3rd (*p* ≤ 0.05), 4th (*p* ≤ 0.001), 5th (*p* ≤ 0.05) and 6th (*p* ≤ 0.001) exposure session. The overall drop of intake between the 4th and the 7th exposure session was found significant (*p* ≤ 0.001), as well as the decrease of daikon intake between the 6th and the 7th exposure (*p* ≤ 0.001). It is important to note here that the intake between the final exposure session was not found significantly higher than the one at the first exposure session for this group. Moreover, it is worth highlighting that while the initial intake for the triangle and stick group was more or less at the same level (45 ± 6 g and 41 ± 6 g, respectively), the initial mean intake for the children that received grated daikon was quite lower at the 1st exposure (27 ± 6 g), although the differences between them were not found statistically significant.

The children who received daikon cut in the shape of a triangle during the intervention showed a significant overall change of intake during the intervention period, with daikon intake of the 7th exposure to be significantly higher than the initial exposure (*p* ≤ 0.001). Moreover, the intake of the first exposure was found significantly lower only than the ones of the 5th (*p* ≤ 0.05) and the 6th session (*p* ≤ 0.001), due to the large drop that took place at the 2nd exposure, which was found to be significant (*p* ≤ 0.05). The consumption of daikon for this group rose continuously from the 2nd to the 6th session, with the changes between the 2nd and the 4th (*p* ≤ 0.001), and the 4th and the 6th (*p* ≤ 0.05) to be significant.

The group that was exposed to daikon cut in sticks demonstrated different kinetics of intake levels during the exposures. Overall, the last exposure’s intake level was significantly higher than the initial exposure (*p* ≤ 0.01), although intake underwent a large decline from the 6th to the 7th exposure (*p* ≤ 0.01). The intake of the 1st session was found significantly lower compared to the intake of the 4th (*p* ≤ 0.01), 5th (*p* ≤ 0.01), 6th (*p* ≤ 0.001) and 7th (*p* ≤ 0.01) exposure.

#### 3.4.1. Exposures 1–4

Benchmarking on the 4th exposure where it was observed that for the stick and grated groups, intake was continuously rising from the 1st to the 4th session. The triangle group showed a drop at the 2nd exposure, which was significant (*p* = 0.014), but followed a steady increase later on.

#### 3.4.2. Exposures 4–7

Except for the triangle group that from the 2nd until the 6th exposure sessions followed a steady increasing pattern, the other two groups changed their intake kinetics after the 4th session. For the grated daikon group, intake dropped at the 5th session to return at rather the same levels and dropped again at the 7th session to reach the lowest intake of the three for this exposure visit. The group that received daikon sticks demonstrated a peak at the 6th exposure, before dropping at the last one. It is important to note that all groups underwent a drop of a different degree at the 7th exposure, compared with the intake that was recorded on the 6th. This drop was smaller for the triangle group, followed by a larger drop for the grated group (*p* ≤ 0.001) and an even larger for the group that received sticks (*p* = 0.002). This decrease was not significant for the triangle group.

### 3.5. Ranking

**Figure 4:**
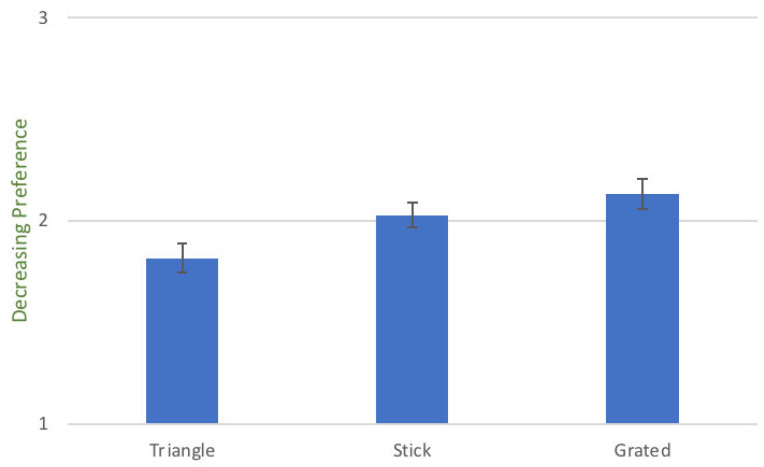
Mean ranking score ± SEM of the three serving styles of daikon on behalf of all the children that participated in the study during the first control session. For ranking data, the evaluation scale used was 1 = most preferred, 2 = second preferred and 3 = least preferred, with the mean value of a highly ranked vegetable to contain the value “1” more times and therefore to have lower score than the least preferred vegetable which contains the value “3” more times. The lowest score (in this case for the triangle-shaped daikon) represents the highest preference on behalf of the children. Similarly, the highest bar represents the least preferred serving style.

Figure 4 shows the mean ranking ± SEM of the three serving styles of daikon, as evaluated by all the children that took part in the study. For the ranking data the scaling is inversed compared to liking scores, as children were ranking as 1 their most preferred serving style, 2 their second preferred serving style and 3 their least preferred serving style. The bar plot shows that triangle was the favourite shape of the children, followed by sticks and then grated daikon. This result is independent of the intervention, as it was measured at baseline level.

**Figure 5:**
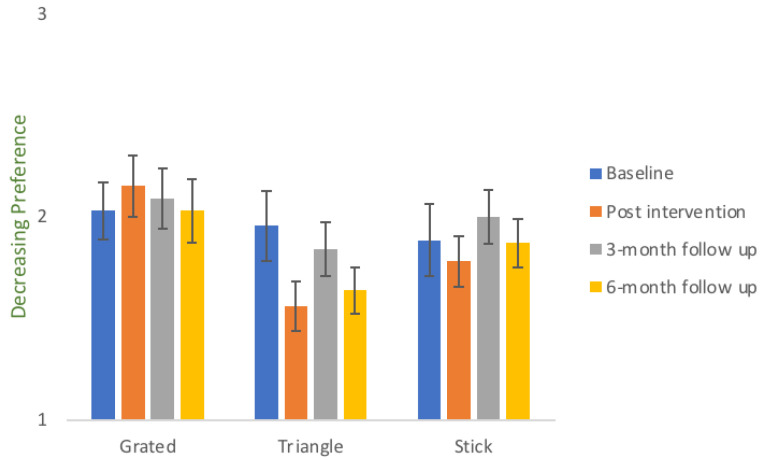
Ranking scores ± SEM for the different shapes of daikon throughout the control sessions. The scores that are illustrated represent the ranking of the respective serving style by the children that were exposed to that particular serving style of the same vegetable during the intervention. For ranking data, the evaluation scale used was 1 = most preferred, 2 = second preferred and 3 = least preferred, with the mean value of a highly ranked vegetable to contain the value “1” more times and therefore to have lower score than the least preferred vegetable which contains the value “3” more times. The lowest scores represent the highest preference. Similarly, the highest bars represent the least preferred serving styles.

Figure 5 illustrates the ranking scores ± SEM for the different shape styles of daikon, as evaluated by the respective control groups on the four control sessions. The ranking of grated daikon is depicted as rated by the exposure group of grated daikon, the ranking of triangle daikon by the children that were exposed to triangles and the ranking of sticks from the children receiving daikon in sticks throughout the exposure sessions. Data analysis did not show any significant differences on the ranking of specific shapes within the exposure groups across the four control sessions. As it is seen in Figure 5, ranking of the particular serving styles does not follow a clear escalating pattern for any of the three shapes.

**Figure 6:**
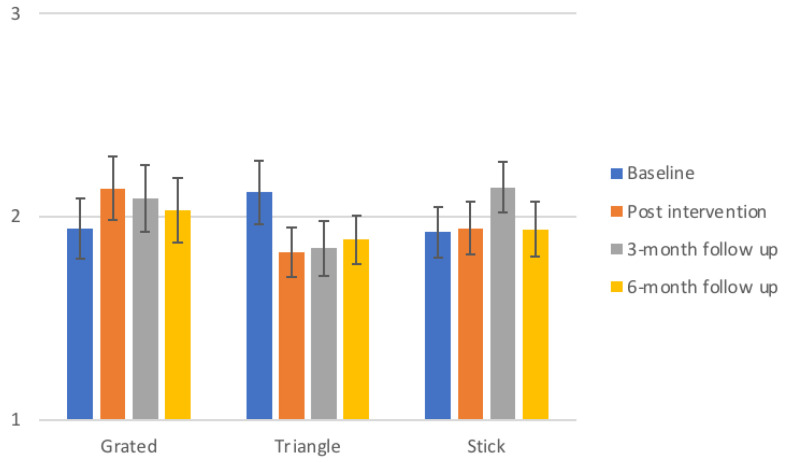
Ranking scores ± SEM for the different shapes of beetroot throughout the control sessions. The scores that are illustrated represent the ranking of the respective serving style by the children that were exposed to that particular serving style of the target vegetable daikon during the intervention. For ranking data 1 = most preferred, 2 = second preferred and 3 = least preferred.

Figure 6 shows the respective ranking information for beetroot serving styles, as rated by the groups that received daikon in the corresponding format during exposures. Similarly to daikon, no significant changes were found in the preference of a particular serving style among the children whom were repeatedly exposed to it. As clearly shown in Figure 6, there is not a clear development of increasing nor decreasing preference towards the exposed shapes.

### 3.6. Generalisation Effect

Daikon is a member of the cabbage family (Brassicaceae), as are broccoli and cauliflower. Beetroot and celeriac were used also to study the possibility of a generalisation effect of the daikon exposures due to different shared components with daikon.

**Figure 7:**
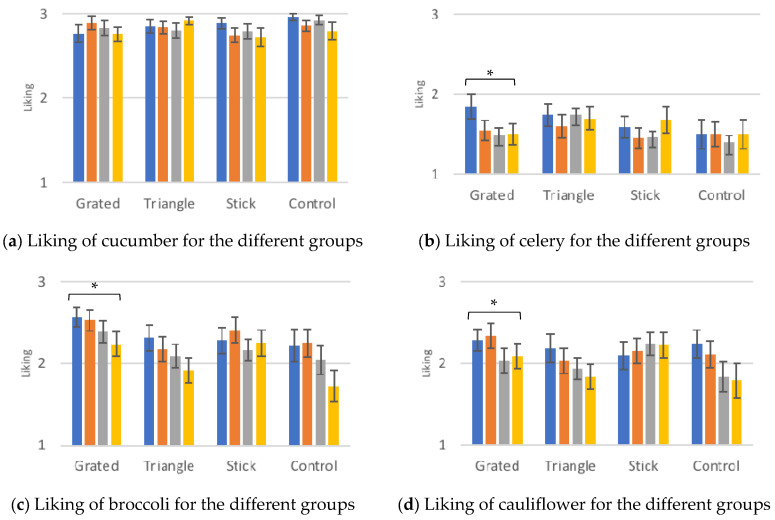

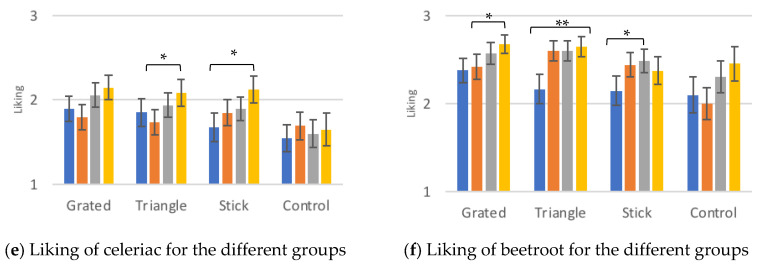
(**a**–**f**) Liking scores ± SEM of the different vegetables measured in the control sessions for each serving style group in order to investigate the presence of transfer effects. The significance of selected differences only for all the groups are depicted. The detailed differences and their statistical significance are given in text. Significance levels: ** *p* ≤ 0.01, * *p* ≤ 0.05.

Cucumber and celery were used as dummy vegetables in order to test the 3-point smiley hedonic scale. Cucumber is a generally liked vegetable, and we can notice that its liking score almost reaches 3, while celery is a generally unfamiliar vegetable, a fact demonstrated also by its liking score in this study, which remains below 2, as shown in Figure 7a–f, which illustrates the liking scores for the dummy (cucumber and celery) and reference (celeriac, broccoli, cauliflower and beetroot) vegetables as reported in the control sessions. Cauliflower was found to be significantly less liked at 6-month follow up (1.8 ± 0.2) compared to baseline (2.2 ± 0.2, *p* ≤ 0.05) and post intervention (2.0 ± 0.2, *p* ≤ 0.05) for the group that was exposed to daikon cut in triangles.

Celery showed a significant decrease in liking from baseline (1.9 ± 0.2) to post intervention (1.5 ± 0.1 *p* ≤ 0.05) and from baseline to the 6-month follow up (1.5 ± 0.1 *p* ≤ 0.05) for the group that received grated daikon. This might just have to do with the fact that celery is a rather disliked vegetable, as it was used mainly to train children for the study and does not share common characteristics with daikon.

Broccoli was significantly less liked at 6-month follow up compared to baseline (*p* ≤ 0.05) from the children who received grated daikon. As broccoli was served as florets for the control sessions, there is not a common ground on colour or shape for this result to be explained by a generalisation effect, considering that it just appeared on the grated daikon group.

Liking of celeriac elevated significantly for the children that received daikon in triangles and in sticks. For the former, liking at 6 months was found remarkably increased compared to post intervention (*p* ≤ 0.05), while for the latter liking at 6 months was significantly higher than baseline liking (*p* ≤ 0.05). Celeriac shares with daikon the same colour, which can be considered “pale” or “bland” and not that “fun” for children to like. This tendency is not though systematic throughout the study; hence such a transfer effect cannot be concluded here.

The liking of beetroot demonstrated several significant differences. For the group that was served grated daikon, the liking between post intervention and the 6-month follow up increased significantly, from 2.4 ± 0.1 to 2.7 ± 0.1 (*p* ≤ 0.05). Similarly, for the triangle group, the differences between baseline liking and post intervention (*p* ≤ 0.05), 3-month follow up (*p* ≤ 0.01) and 6-month follow up (*p* ≤ 0.01), were all statistically significant. Lastly, liking for beetroot increased significantly also for children that were served daikon in sticks, from baseline to 3-month follow up (*p* ≤ 0.05).

The children that participated in the study were exposed to beetroot more than the other reference vegetables, due to the ranking test of the different shapes that was performed in the frame of every control session. Beetroot was presented to the children in exactly the same serving styles as daikon: round, grated, triangle and stick. Ranking of beetroot shapes were shown to be independent from the exposure to the particular shapes of daikon and taking into account that beetroot does not share a colour or taste component with daikon, this increase in liking of beetroot is probably not a result of generalisation effect. This result would be rather expected due to the higher exposure to this vegetable and in combination with the results derived from the ranking data, this study cannot conclude on a generalisation effect to beetroot due to the repeated exposure to daikon. The increase can be observed in all groups, including the control, showing that this infrequent exposure to beetroot contributed to an improvement of its liking. It is worthwhile to note at this point that beetroot is generally characterised as a sweeter vegetable, as shown also in the sensory profiling analysis, hence it might be innately more acceptable to children.

### 3.7. Sensory Profiling

The two taste attributes of salt and umami were excluded from the data, and shown not to be significant on a 3-way ANOVA. The 19 attributes that remained were all included in the following analysis as they were found to have a significant effect. The panel members were found to successfully differentiate the samples on the used attributes with an acceptable replication. The 19 attributes were found to be dependable for the analysis by PCA.

**Figure 8:**
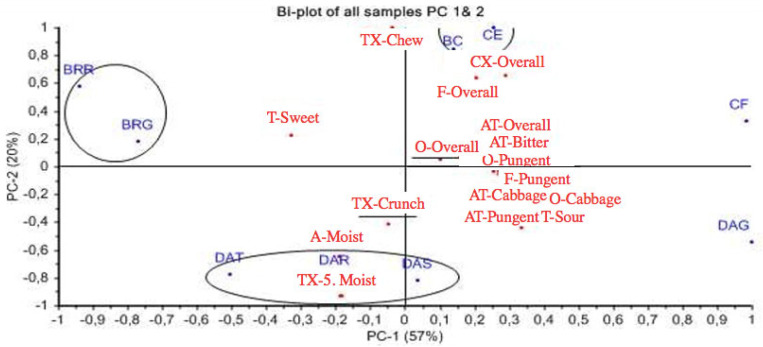
Bi-plot of all samples PC1 and PC2. Abbreviations of samples: BRG: Beetroot grated, BRR: Beetroot round, BC: Broccoli, CF: Cauliflower, CE: Celeriac, DAG: Daikon grated, DAR, Daikon round, DAS, Daikon stick, DAT, Daikon triangle. Abbreviations of attributes: O: Odour, A: Appearance, TX: Texture, T: Basic Taste, F: Flavour, AT: Aftertaste, CX: Complexity.

Figure 8 portrays PC1 and PC2, which explain most of the variance (77%), with PC1 to cover for the 57%. PC1 discriminates the round and grated beetroot from grated Daikon and cauliflower, with the former to be characterized mainly with sweet taste and the latter with pungency and notes of cabbage. On the right side of the plot are the clustered attributes, with the main elements to bitter, pungent and cabbage-like qualities. These attributes are: Odour-pungent (O-Pungent), Odour-cabbage (O-cabbage), Aftertaste-pungent (AT-Pungent), Aftertaste-cabbage (AT-Cabbage), Aftertaste-bitter (AT-Bitter), Flavour-pungent (F-pungent), Flavour-cabbage (F-Cabbage), Taste-sour (T-Sour) and Taste-bitter (T-Bitter).

**Figure 9:**
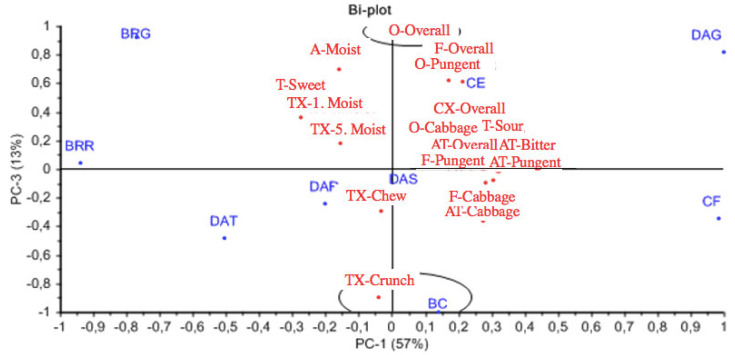
Bi-plot of all samples, PC1 and PC3. Above celeriac are found the attributes F-Overall and O-Pungent, while below celeriac are clustered the following: F-Pungent, AT-Overall, AT-Bitter, AT-Pungent, O-Cabbage, T-Sour, T-Bitter and CX-Overall. Abbreviations of samples: BRG: Beetroot grated, BRR: Beetroot round, BC: Broccoli, CF: Cauliflower, CE: Celeriac, DAG: Daikon grated, DAR, Daikon round, DAS, Daikon stick, DAT, Daikon triangle. Abbreviations of attributes: O: Odour, A: Appearance, TX: Texture, T: Basic Taste, F: Flavour, AT: Aftertaste, CX: Complexity.

Figure 9 shows a bi-plot of PC1 and PC3. The 13% of the variance that is explained by PC3 mainly concerns the crunchiness in texture and the overall odour intensity. Broccoli is characterised as crunchy and it is distinguished from celeriac, which is described by a pungent and intense odour. Pungency, cabbage and overall intensity remain to be attributes of cauliflower and grated daikon, while beetroots continue to be on the sweet side. Grated beetroot is characterised more by overall odour intensity, which is observed also for grated daikon, while round beetroot is considered crunchier.

The results from sensory profiling revealed a difference between the grated Daikon compared to the stick, round and triangle. The grated format was found to be more similar to cabbage, with a more intense odour and pungent flavour and aftertaste than the other three formats that were perceived similar and characterised more as sweet, crunchy and moist. Grated daikon was considered to have a bitter and sour taste and be less sweet than round daikon and had more common flavour components with cauliflower than the other three styles, while they were described differently in terms of texture. The pungency of grated daikon was found similar to celeriac, which has also a very intense odour. Celeriac was shown to be comparable to broccoli in terms of the effort needed to chew, while broccoli was characterised as crunchier than round daikon.

Beetroot was found to have divergent taste attributes from the other vegetables, as it was characterised as sweet. Nevertheless, in a level of texture, grated beetroot was found to be more comparable to grated daikon and round beetroot closer to round daikon.

## 4. Discussion

### 4.1. Age and Gender

For this study, the impact of serving style on liking, preference and intake of daikon was independent of age and gender. Both factors were included as fixed factors in the models run for intake and liking but they were not statistically significant. The model run for the ranking data was reduced to a simpler one, as the fixed factors of age and gender were not found significant. This is in line with previous studies [37,75,99].

### 4.2. Ranking of Different Shapes

The initial ranking of the different styles of daikon showed triangles to be the most preferred shape for the children that took part in the study, with sticks to come second and grated last. This is in line with the study of Olsen et al. [68], which suggested a preference for vegetables cut in special shapes. Moreover, the above is in agreement with the sensory profiling results, that showed a more intense flavour and pungency for grated daikon, while a triangle was perceived sweeter and crunchier, qualities that are generally preferred by children.

Although daikon shares sensory characteristics with known vegetables, it is not part of a typical Danish diet and thus is considered as novel, particularly for children [45]. As discussed in the article of Guiné et al., consumers link traditional foods with habits, such as products they consume frequently and that are integrated in their daily lives, while they associate them to a specific sensory profile [100]. Nacef et al. demonstrated that consumers that are familiar to a food rely on parameters such as aroma, taste and texture for their food choice, while unfamiliar consumers tend to base their choice on appearance cues [101]. Therefore, it makes sense for children to express a preference for the special shapes when it comes to a novel vegetable.

Nevertheless, the comparison results from the ranking data did not find any significant differences over preference of a specific serving style on behalf of the groups that received the respective styles across the duration of the study. This indicates that while exposure itself can increase the acceptance of a novel vegetable, the exposure on a particular serving style of the vegetable does not limit the acceptance of the vegetable overall.

### 4.3. Serving Style Effect

In this study it is not clear which particular shape should be preferred towards increasing the acceptance and intake of a novel vegetable, as all shapes were found to be effective in the long-term, with triangles and sticks performing faster and rather better during the intervention. Children who received triangles increased their overall intake steadily after the 2nd exposure session, while children who received sticks were demonstrating a clear increasing trend until the last exposure session, undergoing a drop that could be attributed to the boredom effect, as it was observed in a different extent in all the exposure groups. This decrease was found to be statistically significant for the groups of sticks (*p* = 0.002) and grated daikon (*p* ≤ 0.001), but not significant for the triangle group. This can be explained by the fact that triangles are a “fun” shape compared to the grated vegetable. The group that received sticks performed rather similarly to the triangle one, increasing remarkably liking from a low value to post intervention, reaching the highest intake levels at 6 months, and achieving the highest intake among all exposures and all groups, at the 6th exposure session. A reason could be that sticks are a shape that might be more familiar to children, thus it might be easier for them to try the novel vegetable in the first place in that format. It has been showed that familiar foods are usually more liked than unfamiliar ones [101] as familiarity reflects the security in terms of what is the served food, reducing anxiety and hesitation towards it [102]. Therefore, it could be hypothesised than when an unknown vegetable, such as daikon, is served in a familiar shape this would result in a higher acceptance and liking level. Nevertheless, for the group that received sticks, both liking and intake were relatively low at baseline, while this serving style was the second preferred at the same time point.

It is interesting to mention that sticks and triangle-shaped daikon have more in common in terms of texture, as well as the fact they can be consumed using the hands, unlike grated daikon that requires a fork. Therefore, this could be another potential driver for children to prefer and consume more of the former shapes. This could be elaborated by the findings of van Eck, who studied the effect of different carrot shapes in eating behaviour, indicating that cutting the vegetable increases the surface area and consequently the mastication effort and eating rate. This might negatively affect vegetable intake compared to serving carrot in larger pieces, which were consumed with more ease and higher eating rates [103]. This is in line with Liem and Russell, who served diced and whole carrots to primary school children and observed a 75% increase of intake in the case of the whole vegetable. In the same logic, the findings of Goh et al. suggest serving a larger unit rather than diced vegetables towards increasing their consumption [104]. It is important to mention that the children that participated in these two studies were older than the ones in the present one, which could affect the chewing ability of larger particles as well as the preference in shape [105]. Moreover, carrot is a familiar and rather liked vegetable which gives it an advantage in intake levels. It would be interesting to perform similar studies for unfamiliar stimuli, like daikon.

### 4.4. Exposure Effect

Liking and intake for daikon were increased significantly for all groups after the seven exposure sessions, including the control group that received just small quantities of the vegetable in the control sessions. Mere exposure strategy was shown to be effective in this study, in line with a variety of previous intervention studies [39,40,106,107,108,109,110,111,112,113,114,115,116,117]. Consequently, it seems that even small quantities and a scarce exposure frequency was sufficient to improve children’s acceptance and consumption of daikon, while the exposure to the vegetable twice per week was found effective for every serving style. In this study, it was found that a minimum number of four exposures were adequate to influence positively the liking of daikon by children. This finding is not exactly in line with Birch and Sullivan [118], and Cooke [38], who suggested an exposure to the vegetable 8–15 times or 5–15 times, respectively in order to be efficient [38,118]. It is worthwhile to mention here that the potential effect of the serving size of the vegetable during the exposure, as in this study the children received a whole 100 g serving, compared to other studies where the quantity of the stimuli was smaller [37,39,40,99,119,120]. This is in line with van Kleef et al., who found that children ate 54% more cucumber when the vegetable was served in a large portion, compared to a smaller one [121]. It would be interesting to investigate such an effect of the serving size on mere exposure efficiency in a future study.

Due to the low response rate of the questionnaire this study could not connect the liking results with the levels of food neophobia of the sample, although we would expect neophobic children to resist in accepting and liking daikon. According to the study of Tuorila et al., food neophobia and unfamiliar sensory properties are potential drivers for a consumer to reject an ethnic food, while it may be accepted if it is widely available [45]. This is in line with the results of our study, as repeated exposure increased the availability of the vegetable.

### 4.5. Limitations

One of the limitations of this study concerns the control group, which was not a “blank” control group but was exposed to small quantities of grated daikon during the control visits. It is obvious that in such an intervention study to an unfamiliar vegetable the control group cannot be exclusively not exposed to the stimuli, as this is needed for the required measurements. Nevertheless, this limitation revealed that even a minimum of a few, scarce exposures to small quantities of daikon can deliver a favourable outcome in children’s intake and liking.

A second limitation of the study was that the maximum intake level during the control visits (200 g) was different compared to the exposure visits (100 g), a fact that potentially complexes the comparison of the amounts of daikon consumed. The children who ate more than one portion of daikon might have caused an increase on the average intake at control visits without this to represent the whole sample. Therefore, it is suggested to account for this issue in future studies.

## 5. Conclusions

The result of this study on whether there is a preferable serving style of a novel stimulus for a more efficient exposure intervention it is not clear. All serving styles were effective at increasing liking and intake of daikon, with the triangles and sticks possibly being slightly superior. The repeated exposure strategy was proven successful with results that remained in the long-term. Intake underwent a decline towards the end of the exposure intervention for all serving styles, but less for triangles. It was demonstrated overall that exposure to a specific shape does not necessarily improve the liking and preference for the specific shape of the same or similar vegetables, although repeated exposure itself generally increased the liking and intake for the vegetable as a whole.

## 6. Implications

The acceptance of a novel food depends on different parameters in terms of behaviour and attitude, that include preference, choice and consumption, as well as a willingness to eat foods and the desired consumption frequency [122].

The aim of this study was to explore the effect of the serving style on the consumption of a novel vegetable. It was demonstrated that the shape of the served vegetable does not significantly influence the preference and intake on behalf of 3–5-year-old children. The effect of repeated exposure was depicted in this study as, independently of the serving style, children consumed and liked the vegetable they were exposed to more. This is an important point for every-day practice, encouraging parents and caregivers to repeatedly present unfamiliar vegetables.

The fact that intake and liking of daikon improved after repeated exposures to every serving style, in practice means that parents and caregivers do not necessarily need to present vegetables cut in a particular shape nor at the same format every time, providing them with flexibility. It seems that even a few infrequent exposures could provide with a benefit with the vegetable being served in every format, with the more “fun” shapes to potentially bring a faster result.

We acknowledge that the above suggestion could be highly influenced by the familial environment. Holley et al. investigated the strategies used by the caregivers towards presenting vegetables to the children, categorising them in active/behavioural methods, passive methods and food manipulations [123]. The same article demonstrated that the caregiver’s personal preferences for vegetables affect whether their children are exposed to specific stimuli, which is in line with other studies [124,125]. Children who are fussier with vegetables have usually been reoffered vegetables less times [125], while it is more likely for mothers who consume more vegetables themselves to reoffer these vegetables to their children because they are concerned, among others, of the food waste related to the rejection of the vegetable [124]. This parameter might be enhanced when the parent is called to cut the vegetable in a specific shape, which could potentially increase waste. It is therefore important for parents and caregivers to keep in mind that as repeated exposure has been demonstrated to be a successful strategy by a previous study [126] and the present study, waste would not be a long-term issue. Moreover, the results of the present study show that every serving style can deliver a positive result, a fact that could reduce parental hesitation to reoffer an unfamiliar vegetable in terms of waste too. It is important to mention that according to Kähkönen et al., it is important to take into account the family influence as a whole. The authors found that the eating habits of the fathers may decrease the preference for fruit and vegetables of their children as they tend to exert a more relaxed eating attitude. This result was shown to be more prominent for strong-tasting vegetables, which are usually the hardest in terms of acceptance [72].

Therefore, it seems that repeated exposure of a vegetable in any serving style is effective and it could have greater results with the appropriate support and paradigm exerted by the family members of the children, as they act as role-models for the children.

## Figures and Tables

**Table 1:** Overview of the content of the different visit types.

Type of Visit	Groups	Test	Vegetable
Pre-test/baseline measurement	All	Individual testing	Familiarity and liking: Broccoli, Cauliflower, Celeriac, Daikon (round), Beetroot (round)Ranking: Daikon and Beetroot grated, triangle and stick
	All	Intake (200 g)	Daikon, round
Exposure twice a week	Sticks	Seven exposures (100 g)	Daikon, in sticks
Exposure twice a week	Triangle	Seven exposures (100 g)	Daikon, in triangles
Exposure twice a week	Grated	Seven exposures (100 g)	Daikon, grated
Post-test	All	Intake (200 g)	Daikon, round
	All	Individual testing	Familiarity and liking: Broccoli, Cauliflower, Celeriac, Daikon (round), Beetroot (round)Ranking: Daikon and Beetroot grated, triangle and stick
3-month follow-up	All	Intake (200 g)	Daikon, round
	All	Individual testing	Familiarity and liking: Broccoli, Cauliflower, Celeriac, Daikon (round), Beetroot (round)Ranking: Daikon and Beetroot grated, triangle and stick
6-month follow-up	All	Intake (200 g)	Daikon, round
	All	Individual testing	Familiarity and liking: Broccoli, Cauliflower, Celeriac, Daikon (round), Beetroot (round)Ranking: Daikon and Beetroot grated, triangle and stick

**Table 2:** Characteristics of the participating children.

	Control	Grated	Triangle	Stick
*n*	50	47	46	42
Girls/boys	23/27	30/17	27/19	19/23
Age (months) ^(1)^	51.75 ± 2.00	55.02 ± 0.86	53.78 ± 2.05	53.77 ± 1.41

^(1)^ Values are expressed as Mean ± SEM.

## Data Availability

Data is not available for sharing.
